# Human T-cell leukemia virus type I (HTLV-I) infection and the onset of adult T-cell leukemia (ATL)

**DOI:** 10.1186/1742-4690-2-27

**Published:** 2005-04-26

**Authors:** Masao Matsuoka

**Affiliations:** 1 Institute for Virus Research, Kyoto University, Kyoto 606-8507, Japan

## Abstract

The clinical entity of adult T-cell leukemia (ATL) was established around 1977, and human T-cell leukemia virus type 1 (HTLV-I) was subsequently identified in 1980. In the 25 years since the discovery of HTLV-I, HTLV-I infection and its associated diseases have been extensively studied, and many of their aspects have been clarified. However, the detailed mechanism of leukemogenesis remains unsolved yet, and the prognosis of ATL patients still poor because of its resistance to chemotherapy and immunodeficiency. In this review, I highlight the recent progress and remaining enigmas in HTLV-I infection and its associated diseases, especially ATL.

## Background

**Figure 1:**
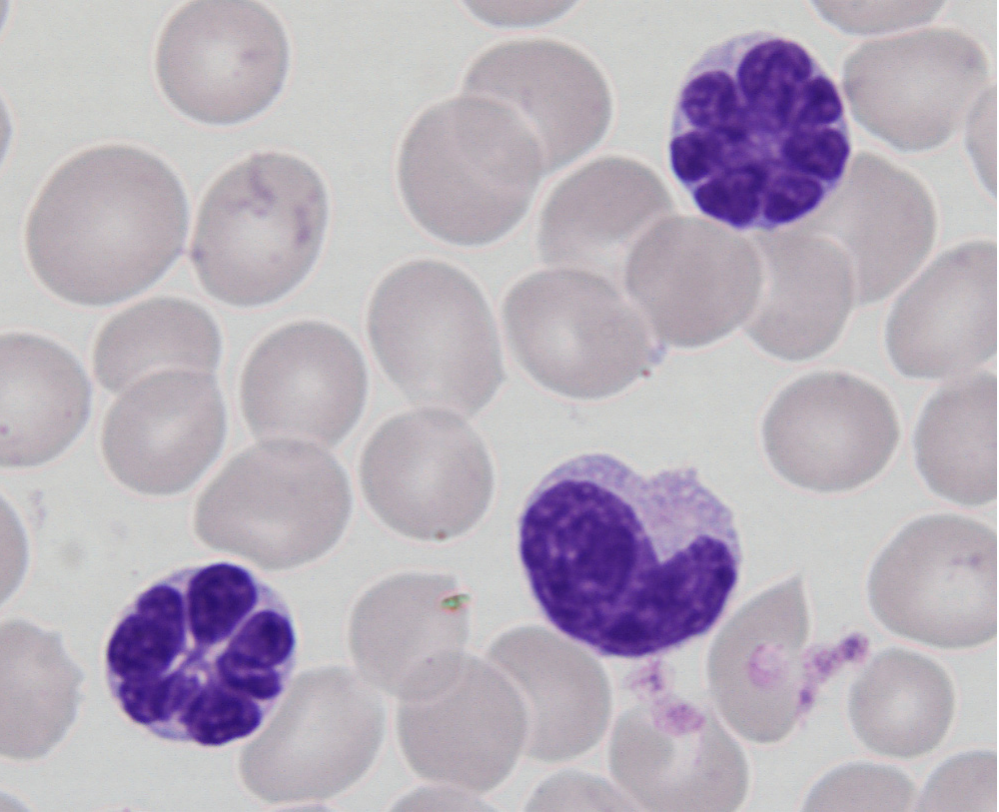
Typical "flower cell" in the peripheral blood of an acute ATL patient. In the peripheral blood of an acute ATL patient, leukemic cells with multilobulated nuclei.

In 1977, Takatsuki et al. reported adult T-cell leukemia (ATL) as a distinct clinical entity [1-3]. This disease is characterized by its aggressive clinical course, infiltrations into skin, liver, gastrointestinal tract and lung, hypercalcemia and the presence of leukemic cells with multilobulated nuclei (flower cell)(Figure 1). In 1980, Poiesz et al. discovered a human retrovirus in a cell line derived from a patient with ATL, and designated it human T-cell leukemia virus type I (HTLV-I) [4,5]. The linkage between ATL and HTLV-I was proven by Hinuma et al., who demonstrated the presence of an antibody against HTLV-I in patient sera [6]. Thereafter, Seiki et al. determined the whole sequence of HTLV-I and revealed the presence of a unique region, designated pX [7]. The pX region encodes several accessory genes, which control viral replication and the proliferation of infected cells [8]. In this review, I describe the recent advances in the field of HTLV-I and ATL research, with particular focus on the mechanism of leukemogenesis and therapeutic aspects.

### 1. History of humans and HTLV-I

HTLV-I is a member of the Deltaretroviruses, which include HTLV-II, bovine leukemia virus and simian T-cell leukemia virus (STLV). The latter two viruses also cause lymphoid malignancies in the host, similar to the case with HTLV-I. HTLV and STLV are thought to originate from common ancestors, and share molecular, virological and epidemiological features. Therefore, they have been designated primate T-cell leukemia viruses (PTLVs). Phylogenetical analyses have revealed that HTLV-Ic first diverged from simian leukemia virus around 50,000 ± 10,000 years ago, while the spread of PTLV-I in Africa is estimated to have occurred at least 27,300 ± 8,200 years ago. Subsequently, HTLV-Ia, which is the most common subtype in Japan, diverged from the African strain 12,300 ± 4,900 years ago [9]. Thus, these viruses have had a long history with humans after the interspecies transmission. In contrast, human immunodeficiency virus type 1 (HIV-1) is thought to originate from simian immunodeficiency virus in chimpanzees (SIV_CPZ_) [10], and the interspecies transmission to humans is estimated to have occurred recently.

### 2. How does HTLV-I spread in humans?

There are approximately 10–20 million HTLV-I carriers in the world [11]. In particular, HTLV-I is endemic in Japan, parts of central Africa, the Caribbean basin and South America. In addition, epidemiological studies of HTLV-I have revealed high seroprevalence rates in Melanesia, Papua New Guinea and the Solomon islands, as well as among Australian aborigines [12]. In Japan, approximately 1.2 million individuals are estimated to be infected by HTLV-I, and more than 800 cases of ATL are diagnosed each year [13]. Moreover, this virus also causes the neurodegenerative disease, HTLV-I-associated myelopathy/tropical spastic paraparesis (HAM/TSP) [14,15]. The cumulative risks of ATL among HTLV-I carriers in Japan are estimated to be about 6.6% for men and 2.1% for women, indicating that most HTLV-I carriers remain asymptomatic throughout their lives [16].

### 3. How does HTLV-I replicate and increase its copy number?

The HTLV-I provirus has a similar structure to other retroviruses: a long terminal repeat (LTR) at both ends and internal sequences such as the *gag*, *pol *and *env *genes. A characteristic of HTLV-I is the presence of the pX region, which exists between *env *and the 3'-LTR. This region encodes several accessory genes, which include the *tax*, *rex*, *p12*, *p21*, *p30*, *p13 *and *HBZ *genes. Among these, the *tax *gene plays central roles in viral gene transcription, viral replication and the proliferation of HTLV-I-infected cells. Tax enhances viral gene transcription from the 5'-LTR via interaction with cyclic AMP responsive element binding protein (CREB). Tax also interacts with cellular factors and activates transcriptional pathways, such as NF-κB, AP-1 and SRF [8,17-20]. For example, activation of NF-κB induces the transcription of various cytokines and their receptor genes, as well as anti-apoptotic genes such as *bcl-xL *and *survivin *[21-23]. The activation of NF-κB has been demonstrated to be critical for tumorigenesis both *in vitro *and *in vivo *[24,25]. On the other hand, Tax variant without activation of NF-κB has also been reported to immortalize primary T-lymphocytes *in vitro *[26], suggesting that mechanisms of immortalization are complex. In addition to NF-κB, activation of other transcriptional pathways such as CREB by Tax should be implicated in the immortalization and leukemogenesis.

Tax also interferes with the functions of p53, p16 and MAD1 [27-30]. These interactions enable HTLV-I-infected cells to escape from apoptosis, and also induce genetic instability. Although inactivation of p53 function by Tax is reported to be mediated by p300/CBP [27,28,31] or NF-κB activation [32], Tax can still repress p53's activity in spite of loss of p300/CBP binding or in cells lacking NF-κB activation [33], indicating the mechanism of p53 inactivation by Tax needs further investigation.

Although Tax promotes the proliferation of infected cells, it is also the major target of cytotoxic T-lymphocytes (CTLs) *in vivo*. Moreover, excess expression of Tax protein is considered to be harmful to infected cells. Therefore, HTLV-I has redundant mechanisms to suppress Tax expression. Rex binds to Rex-responsive element (RxRE) in the U3 and R regions of the 3'-LTR, and enhances the transport of the unspliced *gag*/*pol *and the singly spliced *env *transcripts. By this mechanism, double-spliced *tax*/*rex *mRNA decreases, resulting in suppressed expression of Tax [34]. On the other hand, p30 binds to *tax*/*rex *transcripts, and retains them in the nucleus [35]. The *HBZ *gene is encoded by the complementary strand of HTLV-I, and contains a leucine zipper domain. HBZ directly interacts with c-Jun or JunB [36], or enhances their degradation [37], resulting in the suppression of Tax-mediated viral transcription from the LTR.

Transforming growth factor-β (TGF-β) is an inhibitory cytokine that plays important roles in development, the immune system and oncogenesis. Since TGF-β generally suppresses the growth of tumor cells, most tumor cells acquire escape mechanisms that inhibit TGF-β signaling, including mutations in its receptor and in the Smad molecules that transduce the signal from the receptor. Tax has also been reported to inhibit TGF-β signaling by binding to Smad2, 3 and 4 or CBP/p300 [38,39]. Inhibition of TGF-β signaling enables HTLV-I-infected cells to escape TGF-β-mediated growth inhibition.

ATL cells have been reported to show remarkable chromosomal abnormalities [40], which should be implicated in the disease progression. Tax has been reported to interact with the checkpoint protein MAD1, which forms a complex with MAD2 and controls the mitotic checkpoint. This functional hindrance of MAD1 by Tax protein causes chromosomal instability, suggesting the involvement of this mechanism in oncogenesis [30]. Recently, Tax has been reported to interact with Cdc20 and activate Cdc20-associated anaphase-promoting complex, an E3 ubiquitin ligase that controls the metaphase-to-anaphase transition, thereby resulting in mitotic abnormalities [41].

In contrast to HTLV-I, HTLV-II promotes the proliferation of CD8-positive T-lymphocytes *in vivo*. Although it was first discovered in a patient with variant hairy cell leukemia, HTLV-II is less likely to have oncogenic properties since there is no obvious association between HTLV-II infections and cancers. Regardless of the homology of their *tax *sequences, the oncogenic potential of Tax1 (HTLV-I Tax) is more prominent than that of Tax2 (HTLV-II Tax). The most striking difference is that Tax2 lacks the binding motif at C-terminal end to PDZ domain proteins, while Tax 1 retains it [42]. When the PDZ domain of Tax1 is added to Tax2, the latter acquires oncogenic properties in the rat fibroblast cell line Rat-1, indicating that this domain is responsible for the transforming activity of HTLV-I [43].

To understand the pleiotropic actions of Tax protein more clearly, transcriptome analyses are essential. The transcriptional changes induced by Tax expression have been studied using DNA microarrays, which revealed that Tax upregulated the expression of the mixed-lineage kinase MLK3. MLK3 is involved in NF-κB activation by Tax as well as NIK and MEKK1 [44]. In addition to transcriptional changes, Tax is also well known to interact with cellular proteins and impair or alter their functions. For example, proteomic analyses of Tax-associated complexes showed that Tax could interact with cellular proteins, including the active forms of small GTPases, such as Cdc42, RhoA and Rac1, which should be implicated in the migration, invasion and adhesion of T-cells, as well as in the activation of the JNK pathway [45].

### 4. How does HTLV-I transmit and replicate *in vivo*?

#### Receptor and transmission of HTLV-I

HTLV-I can infect various types of cells, such as T-lymphocytes, B-lymphocytes, monocytes and fibroblasts [46]. Glucose transporter 1 (GLUT-1) has been identified as a receptor for HTLV-I and this receptor is ubiquitously expressed on cell surfaces [47]. However, the HTLV-I provirus is mainly detected in CD4-positive lymphocytes, with about 10% in CD8-positive T-lymphocytes [48]. This situation possibly arises because Tax mainly induces the increase of CD4-positive T-lymphocytes *in vivo *by enhanced proliferation and suppressed apoptosis.

In HTLV-I-infected individuals, no virions are detected in the serum. In addition, the infectivity of free virions is very poor compared with that of infected cells. These findings suggest that HTLV-I is spread by cell-to-cell transmission, rather than by free virions. *In vitro *analyses of HTLV-I-infected cells revealed that HTLV-I-infected cells form "virological synapses" with uninfected cells. Contact between an infected cell and a target cell induces the accumulation of the viral proteins Gag and Env, viral RNA and microtubules, and the viral complex subsequently transfers into the target cell [49]. HTLV-I also spreads in a cell-to-cell manner via such virological synapses *in vivo*.

HTLV-I is mainly transmitted via three routes: 1) mother-to-infant transmission (mainly through breast feeding) [50]; 2) sexual transmission (mainly from male-to-female); and 3) parenteral transmission (blood transfusion or intravenous drug use) [12]. In either route, HTLV-I-infected cells are essential for transmission. This was supported by the findings that fresh frozen plasma from carriers did not cause transmission [51] and freeze-thawing of breast milk reduced vertical transmission [52].

#### Provirus load and transmission

The provirus load varies more than 1000-fold among asymptomatic carriers [53]. Since most infected cells are considered to have one copy of the provirus, the provirus load indicates the percentage of infected cells among lymphocytes. The provirus load is relatively constant during the latent period [53]. Analysis of naive individuals who seroconvert after marrying an HTLV-I-seropositive spouse demonstrated that the proviral gp46 sequences are identical among married couples. This finding confirmed that HTLV-I is transmitted from a seropositive individual to an uninfected spouse. The provirus loads frequently differ between couples despite infection by the same HTLV-I virus, indicating that the number of infected cells is determined by host factors rather than virus itself [54].

Why does HTLV-I increase the number of infected cells by the pleiotropic actions of Tax? The provirus load in peripheral blood mononuclear cells (PBMCs) is well correlated with that in breast milk, and a higher provirus load in breast milk increases the risk of vertical transmission of HTLV-I [55,56]. Similarly, a higher provirus load in PBMCs may be associated with a higher risk of sexual transmission. Thus, an increase in the number of infected cells by the actions of accessory genes, especially *tax*, facilitates transmission. Therefore, HTLV-I has strategies that increase the number of HTLV-I-infected cells via the action of accessory gene products, thereby increasing the chance of transmission.

#### Clonal expansion of HTLV-I-infected cells

After HTLV-I infection, viral proteins such as Tax protein promote the proliferation of infected cells and also inhibit apoptosis by their pleiotropic actions. Since the HTLV-I provirus is randomly integrated into the host genome, the identification of integration sites enables to identify each infected clone, and to trace the kinetics of infected cells *in vivo*. Analyses using inverse PCR, which can identify the integration sites of the HTLV-I provirus, revealed that the proliferation of infected cells is oligoclonal, and that infected cells persistently survive *in vivo *[57-59]. Importantly, such clonal expansion in carriers is directly associated with the onset of ATL [60]. Thus, the viral strategies to increase the number of HTLV-I-infected cells work efficiently in most carriers without any adverse effects. However, the increased number of infected cells causes an excess immune reaction, leading to inflammatory diseases, HAM/TSP, infective dermatitis [61] or HTLV-I-associated uveitis [62]. Moreover, such prolonged proliferation of infected CD4-positive T-lymphocytes results in the onset of ATL in some carriers after a long latent period.

#### Inactivation of Tax expression in ATL cells

**Figure 2:**
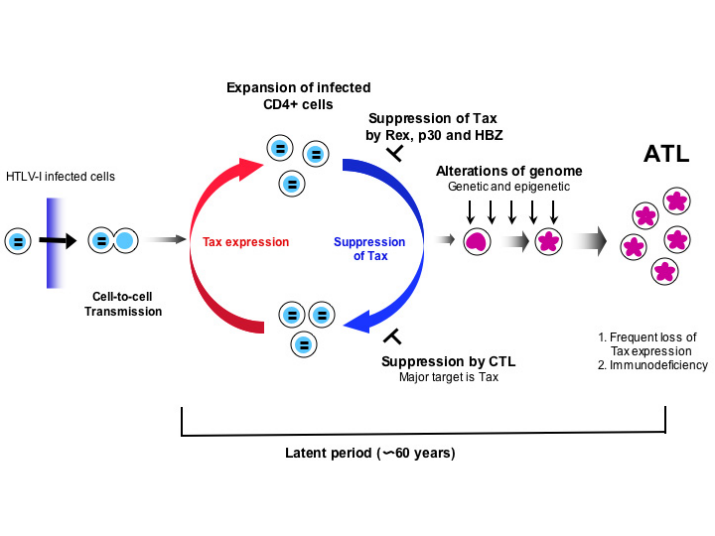
Natural course of HTLV-I infection to onset of ATL. HTLV-I is transmitted via three routes, and infected cells are necessary in all three. After infection, HTLV-I promotes clonal proliferation of infected cells by pleiotropic actions of Tax. Tax expression is suppressed by viral accessory gene products, such as Rex, p30 and HBZ proteins. Proliferation of HTLV-I infected cells is controlled by cytotoxic T-cells *in vivo*. After a long latent period, ATL develops in about 5% of asymptomatic carriers. The expression of Tax is inactivated by several mechanisms, suggesting that Tax is not necessary in this stage. Alternatively, alternations in the host genome accumulate during the latent period, finally leading to onset of ATL.

As mentioned above, Tax expression confers advantages and disadvantages on HTLV-I-infected cells. Although the proliferation of infected cells is promoted by Tax expression, CTLs attack the Tax-expressing cells since Tax is their major target [63]. In HTLV-I-infected cells, Rex, p30 and HBZ suppress Tax expression. On the other hand, loss of Tax expression is frequently observed in leukemic cells. Three mechanisms have been identified for inactivation of Tax expression: 1) genetic changes of the *tax *gene (nonsense mutations, deletions or insertions) [64,65]; 2) DNA methylation of the 5'-LTR [65,66]; and 3) deletion of the 5'-LTR (Figure 2) [67]. Among fresh leukemic cells isolated from ATL patients, about 60% of cases do not express the *tax *gene transcript. Interestingly, ATL cells with genetic changes of the *tax *gene expressed its transcripts, suggesting that ATL cells do not silence the transcription when the *tax *gene is abortive [65]. Loss of Tax expression gives ATL cells advantage for their survival since they can escape from CTLs.

#### Longer lifespan of HTLV-I-infected cells and cancer

Lymphoid malignancy with a T-cell origin is rare compared with B-cell malignancy. ATL shares hematological, pathological and immunological features with cutaneous T-cell lymphoma (CTCL; Sezary syndrome and Mycosis fungoides). The frequency of CTCL in Japan is estimated to be one/million/year. On the other hand, the frequency of ATL among carriers is estimated to be 1000/million/year. From these data, HTLV-I infection is estimated to increase the risk of T-cell malignancy by up to 1000-fold in carriers.

HTLV-I infection confers a long lifespan on the infected cells due to the pleiotropic actions of Tax, resulting in increased numbers of infected cells. Such infected cells are essential for the transmission of HTLV-I. This strategy to increase the number of infected cells *in vivo *is thought to increase the incidence of cancer in T-cells. What is the mechanism for this oncogenesis? DNA methylation is known to be associated with aging. Some genes are hypermethylated in older people, indicating that DNA hypermethylation is a physiological phenomenon in some genes. Under normal circumstances, T-lymphocytes survive for several years, and long-lived T-lymphocytes with disordered methylation should be replaced. However, HTLV-I-infected T-cells are considered to survive and accumulate abnormal methylation. The process of oncogenesis is similar to that of evolution [68]. The infected cells that are suitable for survival should be selected *in vivo*, and epigenetic and genetic changes of the genome play critical roles in this selection. Accumulating alterations of the host genome transform the HTLV-I-infected cells into ATL cells, and also enable ATL cells to proliferate in the absence of Tax expression (Figure 2). In the provirus, DNA methylation of the 5'-LTR silences viral transcription in leukemic cells, which facilitates the escape of ATL cells from the host immune system [65].

### 5. Somatic alterations in ATL cells

As described, some ATL cells can proliferate without functional Tax protein, suggesting that somatic (genetic and epigenetic) alterations cause transcriptional or functional changes to the host genes. The *p53 *gene is frequently mutated in various cancers, and these mutations are associated with disease progression and a poor prognosis. The mutation rate of the *p53 *gene in ATL cells has been reported to be 36% (4/11) and 30% (3/10) [69-71]. The *p16 *gene is an inhibitor of cyclin-dependent kinase 4/6, and blocks the cell cycle. Genetic changes in this gene (deletion in most cases) have been described in many types of cancer cells. Deletion of the *p16 *gene has also been reported in ATL cells [72]. Moreover, DNA methylation of the promoter region of the *p16 *gene is also implicated in the suppression of p16 [73]. In addition, genetic changes in the *p27*^*KIP*1^, *RB1/p105 *and *RB2/p130 *genes have been reported in ATL, although they are relatively rare: 2/42 (4.8%) for the *p27*^*KIP*1 ^gene; 2/40 (5%) for the *RB1/p105 *gene; and 1/41 (2.4%) for the *RB2/p130 *gene) [74]. The fact that higher frequencies of genetic changes in these tumor suppressor genes are observed among aggressive forms of ATL suggests that such genetic changes are implicated in disease progression.

Fas antigen was the first identified death receptor. It transduces the death signal by binding of its ligand, Fas ligand (FasL). ATL cells highly express Fas antigen on their cell surface [75], and are highly susceptible to death signals mediated by agonistic antibodies to Fas antigen, such as CH-11. Genetic changes of *Fas *gene in ATL cells, which confer resistance to the Fas-mediated signal, have been reported [76,77]. Normal activated T-lymphocytes express FasL as well as Fas antigen. Apoptosis induced by autocrine mechanisms is designated activation-induced cell death (AICD) and this controls the immune response [78]. Although ATL cells express Fas antigen, they do not produce FasL, thereby enabling ATL cells to escape from AICD. Attempts to isolate hypermethylated genes from ATL cells identified the *EGR3 *gene as a hypermethylated gene compared to PBMCs from carriers [79]. EGR3 is a transcriptional factor with a zinc finger domain, that is essential for transcription of the *FasL *gene [80]. The finding that *EGR3 *gene transcription is silenced in ATL cells could account for the loss of FasL expression, and the escape of ATL cells from AICD. Thus, alterations of the *Fas *(genetic) and *EGR3 *(epigenetic) genes are examples of ATL cell evolution *in vivo*.

Disordered DNA methylation has been identified in the genome of ATL cells compared with that of PBMCs from carriers: hypomethylation is associated with aberrant expression of the *MEL1S *gene [81], while hypermethylation silences transcription of the *p16 *[73], *EGR3 *and *KLF4 *genes as well as many others [79]. It is reasonable to consider that other currently unidentified genes are involved in such alterations of the genome in ATL cells, and play roles in leukemogenesis.

Transcriptome analyses using DNA microarrays have revealed transcriptional changes that are specific to ATL cells. Among 192 up-regulated genes, the expressions of the *tumor suppressor in lung cancer 1 (TSLC1)*, *caveolin 1 *and *prostaglandin D2 synthase *genes were increased more than 30-fold in fresh ATL cells compared with normal CD4+ and CD4+, CD45RO+ T-cells [82]. TSLC1 is a cell adhesion molecule that acts as a tumor suppressor in lung cancer. Although TSLC1 is not expressed on normal T-lymphocytes, all acute ATL cells show ectopic TSLC1 expression. Enforced expression of TSLC1 enhances both the self-aggregation and adhesion abilities to vascular endothelial cells in ATL cells. Thus, TSLC1 expression is implicated in the adhesion or infiltration of ATL cells. By screening a retrovirus cDNA library from ATL cells, a gene with oncogenic potency was identified in NIH3T3 cells, and designated the *Tgat *gene [83]. Ectopic expression of the *Tgat *gene is observed in aggressive forms of ATL, and *in vitro *experiments showed that its expression is associated with an invasive phenotype.

### 6. Immune control of HTLV-I infection

The host immune system, especially the cellular response, against HTLV-I exerts critical control over virus replication and the proliferation of infected cells [84]. CTLs against the virus have been extensively studied, and Tax protein was found to be the dominant antigen recognized by CTLs *in vivo *[63]. HTLV-I-specific CD8-positive CTLs are abundant and chronically activated. The paradox is that the frequency of Tax-specific CTLs is much higher in HAM/TSP patients than in carriers. Since the provirus load is higher in HAM/TSP patients, this finding suggests that the CTLs in HAM/TSP cannot control the number of infected cells. One explanation for this is that the CTLs in HAM/TSP patients show less efficient cytolytic activity toward infected cells, whereas CTLs in carriers can suppress the proliferation of infected cells [85]. Hence, the gene expression profiles of circulating CD4+ and CD8+ lymphocytes were compared between carriers with high and low provirus loads. The results revealed that CD8+ lymphocytes from individuals with a low HTLV-1 provirus load show higher expressions of genes associated with cytolytic activities or antigen recognition than those from carriers with a high provirus load [86]. Thus, CD8+ T-lymphocytes in individuals with a low provirus load successfully control the number of HTLV-I-infected cells due to their higher CTL activities. Thus, the major determinant of the provirus load is thought to be the CTL response to HTLV-I.

As mentioned above, the provirus load is considered to be controlled by host factors. Considering that the cellular immune responses are critically implicated in the control of HTLV-I infection, human leukocyte antigen (HLA) should be a candidate for such a host genetic factor. From analyses of HAM/TSP patients and asymptomatic carriers, HLA-A02, and Cw08 are independently associated with a lower provirus load and a lower risk of HAM/TSP. In addition, polymorphisms of other genes (*TNF-α, SDF-1, HLA-B54, HLA-DRB-10101 *and *IL-15*) are also associated with the provirus load, although their associations are not as significant compared with HLA-A02, and Cw08 [87,88]. Regarding the onset of ATL, only a polymorphism of *TNF-α *gene was reported to show an association [89]. However, familial clustering of ATL cases is a well-known phenomenon, strongly suggesting that genetic factors are implicated in the onset of ATL [90-92].

Spontaneous remission is more frequently observed in patients with ATL than those with other hematological malignancies [90,93]. Usually, this phenomenon is associated with infectious diseases, suggesting that immune activation of the host enhances the immune response against ATL cells. If the immune response against HTLV-I is implicated in spontaneous remission, this suggests the possibility of immunotherapy for ATL patients by the induction of an immune response to HTLV-I [94], for example via antigen-stimulated dendritic cells.

Immunodeficiency in ATL patients is pronounced, and results in frequent opportunistic infections by various pathogens, including *Pneumocystis carinii*, cytomegalovirus, fungus, *Strongyloides *and bacteria, due to the inevitable impairment of the T-cell functions [95]. To a lesser extent, impaired cell-mediated immunity has also been demonstrated in HTLV-I carriers [96]. Such immunodeficiency in the carrier state may be associated with the leukemogenesis of ATL by allowing the proliferation of HTLV-I-infected cells. A prospective study of HTLV-I-infected individuals found that carriers who later develop ATL have a higher anti-HTLV-I antibody and a low anti-Tax antibody level for up to 10 years preceding their diagnosis. This finding indicates that HTLV-I carriers with a higher anti-HTLV-I titer, which is roughly correlated with the HTLV-I provirus load, and a lower anti-Tax reactivity may be at the greatest risk of developing ATL [97]. The anti-HTLV-I antibody and soluble IL-2 receptor (sIL-2R) levels are correlated with the HTLV-I provirus load [53], and a high antibody titer and high sIL-2R level are risk factors for developing ATL among carriers [98]. Taken together, these findings suggest that a higher proliferation of HTLV-I-infected cells and a low immune response against Tax may be associated with the onset of ATL. Given these findings, potentiation of CTLs against Tax via a vaccine strategy may be useful for preventing the onset of ATL [99].

EBV-associated lymphomas frequently develop in individuals with an immunodeficient state associated with transplantation or AIDS. This has also been reported in an ATL patient [100]. Does such an immunodeficient state influence the onset of ATL? Among 24 patients with post-transplantation lymphoproliferative disorders (PT-LPDs) after renal transplantation in Japan, 5 cases of ATL have been reported. Considering that most PT-LPDs are of B-cell origin in Western countries, this frequency of ATL in Japan is quite high. Although the high HTLV-I seroprevalence is due to blood transfusion during hemodialysis, the immunodeficient state during renal transplantation apparently promotes the onset of ATL [101]. In addition, when experimental allogeneic transplantation was performed to 12 rhesus monkeys and immunosuppressive agents (cyclosporine, prednisolone or lymphocyte-specific monoclonal antibodies) were administered to prevent rejection, 4 of the 7 monkeys that died during the experiment showed PT-LPDs. Importantly, the STLV provirus was detected in all PT-LPD samples [102]. These observations emphasize that transplantation into HTLV-I-infected individuals or from HTLV-I positive donors require special attention.

Although the mechanism of immunodeficiency remains unknown, some previous reports have provided important clues. One mechanism for immunodeficiency is that HTLV-I infects CD8-positive T-lymphocytes, which may impair their functions [48]. Indeed, the immune response against Tax via HTLV-I-infected CD8-positive T-cells renders these cells susceptible to fratricide mediated by autologous HTLV-I-specific CD8-positive T-lymphocytes [103]. Fratricide among virus-specific CTLs could impair the immune control of HTLV-I. Another mechanism for immunodeficiency is based on the observation that the number of naive T-cells decreases in individuals infected with HTLV-I via decreased thymopoiesis [48]. In addition, CD4+ and CD25+ T-lymphocytes are classified as immunoregulatory T-cells that control the host immune system. Regulatory T-cells suppress the immune reaction via the expression of immunoregulatory molecules on their surfaces. The *FOXP3 *gene has been identified as a master gene that controls gene expressions specific to regulatory T-cells. *FOXP3 *gene transcription can be detected in some ATL cases (10/17; 59%) [104]. Such ATL cells are thought to suppress the immune response via expression of immunoregulatory molecules on their surfaces, and production of immunosuppressive cytokines.

### 6. Pathogenesis of HTLV-I infection

ATL cells are derived from activated helper T-lymphocytes, which play central roles in the immune system by elaborating cytokines and expressing immunoregulatory molecules. ATL cells are known to retain such features, and this cytokine production or surface molecule expression may modify the pathogenesis.

ATL is well known to infiltrate various organs and tissues, such as the skin, lungs, liver, gastrointestinal tract, central nervous system and bone [95]. This infiltrative tendency of leukemic cells is possibly attributable to the expressions of various surface molecules, such as chemokine receptors and adhesion molecules. Skin-homing memory T-cells uniformly express CCR4, and its ligands are thymus and activation-regulated chemokine (TARC) and macrophage-derived chemokine (MDC). CCR4 is expressed on most ATL cells. In addition, TARC and MDC are expressed in skin lesions in ATL patients. Thus, CCR4 expression should be implicated in the skin infiltration [105]. On the other hand, CCR7 expression is associated with lymph node involvement [106]. OX40 is a member of the tumor necrosis factor family, and was reported to be expressed on ATL cells [107]. It was also identified as a gene associated with the adhesion of ATL cells to endothelial cells by a functional cloning system using a monoclonal antibody that inhibited the attachment of ATL cells [108]. Thus, OX40 is also implicated in the cell adhesion and infiltration of ATL cells.

**Figure 3:**
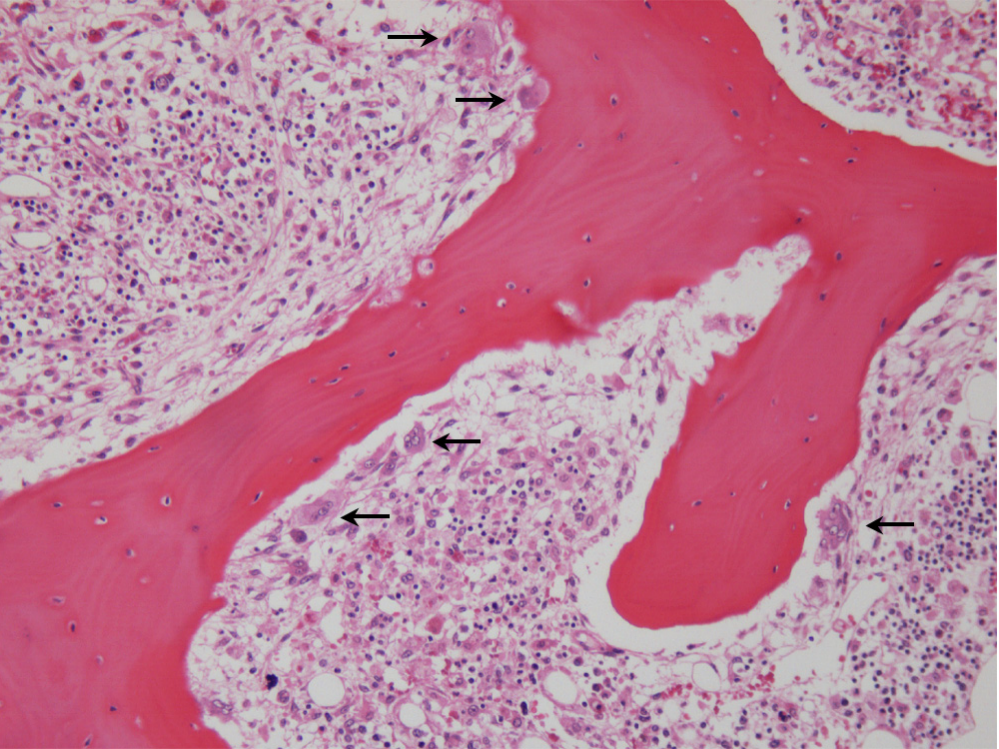
Increased number of osteoclasts in the bone of a hypercalcemic ATL patient. In a hypercalcemic patient, the number of osteoclast (arrows) increased in the bone, which accelerated bone resorption.

Hypercalcemia is frequently complicated in patients with acute ATL (more than 70% during the whole clinical course) [109]. In hypercalcemic patients, the number of osteoclasts increases in the bone (Figure 3). RANK ligand, which is expressed on osteoblasts, and M-CSF act synergistically on hematopoietic precursor cells, and induce the differentiation into osteoclasts [110]. ATL cells from hypercalcemic ATL patients express RANK ligand, and induced the differentiation of hematopoietic stem cells into osteoclasts when ATL cells were co-cultured with hematopoietic stem cells [111]. In addition, the serum level of parathyroid hormone-related peptide (PTH-rP) is also elevated in most of hypercalcemic ATL patients. PTH-rP indirectly increases the number of osteoclasts, as well as activating them [112,113], which is also implicated in mechanisms of hypercalcemia.

### 7. Treatment of ATL – the remaining mission and challenges

Regardless of intensive chemotherapies, the prognosis of ATL patients has not so improved. The median survival time of acute or lymphoma-type ATL was reported to be 13 months with the most intensive chemotherapy [114]. Such a poor prognosis might be due to: 1) the resistance of ATL cells to anti-cancer drugs; and 2) the immunodeficient state and complicated opportunistic infections as described above. Regarding the resistance to anti-cancer drugs, one mechanism is the activated NF-κB pathway in ATL cells [115], which increases the transcription of anti-apoptotic genes such as *bcl-xL *and *survivin*. A proteasome inhibitor, bortezomib, is currently used for the treatment of multiple myeloma. One of its mechanisms is suppression of the NF-κB pathway by inhibiting the proteasomal degradation of IκB protein. Several groups have shown that bortezomib is effective against ATL cells both *in vitro *and *in vivo *[116-119]. Since the sensitivity to bortezomib is well correlated with the extent of NF-κB activation, the major mechanism of the anti-ATL effect is speculated to be inhibition of NF-κB. In addition, an NF-κB inhibitor has also been demonstrated to be effective against ATL cells [120].

During chemotherapy for ATL, chemotherapeutic agents worsen the immunodeficient state of ATL patients. In this regard, antibody therapy against ATL cells has advantages due to its decreased adverse effects. A humanized monoclonal antibody to CD25 has been clinically administered to patients with ATL [121,122]. In addition, a monoclonal antibody to CD2 is at the preclinical stage [123]. As described above, most ATL cells express CCR4 antigen on their surfaces, and a humanized antibody against CCR4 is being developed as an anti-ATL agent [124].

Advances in the treatment of ATL were brought about by allogeneic bone marrow or stem cell transplantation [125,126]. Absence of graft-versus-host disease (GVHD) was linked with relapse of ATL, suggesting that GVHD or graft-versus-ATL may be implicated in the clinical effects of allogeneic stem cell transplantation [125]. Furthermore, 16 patients with ATL, who were over 50 years of age, were treated with allogeneic stem cell transplantation with reduced conditioning intensity (RIST) from HLA-matched sibling donors [127]. Among 9 patients in whom ATL relapsed after transplantation, 3 achieved a second complete remission after rapid discontinuation of cyclosporine A. This finding strongly suggests the presence of a graft-versus-ATL effect in these patients. In addition, Tax peptide-recognizing cells were detected by a tetramer assay (HLA-A2/Tax 11–19 or HLA-A24/Tax 301–309) in patients after allogeneic stem cell transplantation [128]. In 8 patients, the provirus became undetectable by real-time PCR. Among these, 2 patients who received grafts from HTLV-I-positive donors also became provirus-negative by real-time PCR after RIST. Since the provirus load is relatively constant in HTLV-I-infected individuals [53], this finding indicates an enhanced immune response against HTLV-I after RIST, which suppresses the provirus load. This may account for the effectiveness of allogeneic stem cell transplantation to ATL. However, Tax expression is frequently lost in ATL cells as described above. Many questions arise, such as whether the *tax *gene status is correlated with the effect of allogeneic stem cell transplantation, and whether the effectiveness of the anti-HTLV-I immune response is against leukemic cells or non-leukemic HTLV-I-infected cells. Nevertheless, these data suggest that potentiation of the immune response against viral proteins such as Tax may be an attractive way to treat ATL patients [94]. Such strategies may enable preventive treatment of high-risk HTLV-I carriers, such as those with familial ATL history, predisposing genetic factors to ATL, a higher provirus load, etc.

### 8. Two human retroviruses – HTLV-I and HIV-1

As described in the first section, HTLV-I has resided in humans for a long time. On the other hand, HIV-1 has only been recently transmitted to humans, probably from chimpanzees. Due to the comparatively small genomic differences between humans and chimpanzees, this virus can quickly adapt to human cells. These two human retroviruses are opposite in many aspects. HIV-1 vigorously replicates *in vivo*, and the maximum production of HIV-1 virions in the body can reach 10^10 ^per day. Since reverse transcriptase is an error-prone enzyme due to its lack of proof-reading activity, it produces about one mistake per replication, resulting in tremendous errors in the proviral sequence during replication. Although most of these variations ruin the virus replication due to nonsense mutations or impairment of viral gene functions, some become capable of replicating under different circumstances such as the presence of anti-HIV drugs and activation of the host immune system. This can account for why HIV-1 acquires resistance against anti-HIV drugs, and escape from CTLs. On the other hand, HTLV-I increases its copy number in two ways, namely replication of HTLV-I itself and the proliferation of HTLV-I-infected cells *in vivo*. Although immune responses (antibodies, CTLs) against viral proteins suggest the presence of active viral replication *in vivo*, most of increased HTLV-I provirus load (the number of infected cells) is considered to be due to proliferation of infected cells since CTLs efficiently eliminate virus-expressing cells. Therefore, there is much less variation in the HTLV-I provirus sequence compared with HIV-1 [129]. However, this strategy by which HTLV-I increases the number of infected cells due to clonal expansion generates unfortunate side effects for both the host and the virus, namely oncogenesis of CD4-positive T-lymphocytes and the development of ATL.
